# Milk Proteins—Their Biological Activities and Use in Cosmetics and Dermatology

**DOI:** 10.3390/molecules26113253

**Published:** 2021-05-28

**Authors:** Kinga Kazimierska, Urszula Kalinowska-Lis

**Affiliations:** Department of Cosmetic Raw Material Chemistry, Faculty of Pharmacy, Medical University of Lodz, Muszyńskiego 1, 90-151 Łódź, Poland; kinga.kazimierska@stud.umed.lodz.pl

**Keywords:** milk, colostrum, casein, β-lactoglobulin, α-lactalbumin, lactoferrin, growth factors, skin, regeneration, antimicrobial, cosmetics

## Abstract

Milk and colostrum have high biological potential, and due to their natural origin and non-toxicity, they have many uses in cosmetics and dermatology. Research is ongoing on their potential application in other fields of medicine, but there are still few results; most of the published ones are included in this review. These natural products are especially rich in proteins, such as casein, β-lactoglobulin, α-lactalbumin, lactoferrin, immunoglobulins, lactoperoxidase, lysozyme, and growth factors, and possess various antibacterial, antifungal, antiviral, anticancer, antioxidant, immunomodulatory properties, etc. This review describes the physico-chemical properties of milk and colostrum proteins and the natural functions they perform in the body and compares their composition between animal species (cows, goats, and sheep). The milk- and colostrum-based products can be used in dietary supplementation and for performing immunomodulatory functions; they can enhance the effects of certain drugs and can have a lethal effect on pathogenic microorganisms. Milk products are widely used in the treatment of dermatological diseases for promoting the healing of chronic wounds, hastening tissue regeneration, and the treatment of acne vulgaris or plaque psoriasis. They are also increasingly regarded as active ingredients that can improve the condition of the skin by reducing the number of acne lesions and blackheads, regulating sebum secretion, ameliorating inflammatory changes as well as bestowing a range of moisturizing, protective, toning, smoothing, anti-irritation, whitening, soothing, and antiaging effects.

## 1. Introduction

Although milk is known to be used as a raw material in the food industry, it is also widely used in the pharmaceutical and cosmetic industries due to its considerable biological potential. It has also been the subject of detailed analyses and discussions of its individual components and their properties [1,2].

Milk contains the nutrients required for the growth and development of the neonate. It contains a colloidal dispersion of specific proteins as micelles, fats organized in emulsified globules, as well as lactose, various minerals, and vitamins in solution [3].

The composition of milk varies between species. The largest differences can be found between the protein content of individual species. Sheep milk and sheep colostrum is distinguished by the highest total protein and fat content, being almost one and a half that of cow or goat milk and colostrum (Table 1).

The initial milk, or colostrum, is produced by the mammary glands during pregnancy and immediately after delivery for about five days [4,5,6]. Colostrum is yellow, has a slightly acidic pH of about 6.4, and a higher density than later milk. Due to its intended use, it has a much higher content of biologically active substances that affect the immune system of neonates.

**Table 1:** Composition of bovine, goat, and sheep colostrum and milk (%) [3,7,8,9,10,11,12,13].

Component	Colostrum			Milk		
	Bovine	Goat	Sheep	Bovine	Goat	Sheep
Lactose	3.6	3.39–4.24 ^a^	3.3	4.6	4.1	4.8; 4.9 ^b^
Minerals	0.9	0.85–0.9 ^a^	0.9	0.7	0.8	0.94; 1 ^b^
Proteins	7.1	3.53–5.69 ^a^	11.8	3.4	2.9	5.5
Fats	5.1	3.88–8.21 ^a^	13	3.7	4.5	6; 7.4 ^b^

^a^ the minimum and maximum values from the test are given; ^b^ given values from two sources.

Bovine colostrum is several times richer in caseins, β-lactoglobulins, α-lactalbumin, immunoglobulins, GMP (glycomacropeptide) lactoferrin, and growth factors than milk. For example, the immunoglobulins content ranges from 20 to 150 g/L in colostrum, but in the milk, only from 0.5 to 1.0 g/L. Only the content of lactoperoxidase and lysozyme remains at a similar level to both raw materials. Of all the types of colostrum presented, goat colostrum is the richest source of β-lactoglobulin. In goat colostrum, as in sheep colostrum, the content of β-lactoglobulin, α-lactalbumin, IgG, IgM, IgA, and lactoferrin is much higher than in milk of the same species. Sheep milk is rich in casein in comparison to goat and bovine milk; its content, equal to 4.6 g/L, is almost twice as high as in goat’s and cow’s milk (2.5 g/L and 2.7–2.8 g/L, respectively) (Table 2). Therefore, colostrum from each of the mentioned species could be used as a raw material supplying proteins, especially those with biologically active properties.

The main source of active molecules of milk consists of casein and whey proteins, which demonstrate a range of antimicrobial, antioxidant, and immunomodulatory properties, among others [14]. Most biologically active proteins need to undergo proteolysis to achieve their full properties [15,16]. Proteolysis naturally occurs in the digestive tract, but it can also be initiated by the enzymes naturally found in milk, as well as by coagulants or lactic acid bacteria, which are often used in the food industry [15].

## 2. Bioactive Proteins of Milk

### 2.1. Casein

It belongs to the family of milk proteins containing phosphorus (phosphoprotein) and sugar (glycoprotein) residues; it consists of about 20 protein components. Caseins precipitate from raw, skimmed milk at a temperature of 20 °C at a pH of 4.6. The proteins of the four casein fractions (α_S1_-, α_S2_-, β-, and κ-casein) account for 80% of the total protein content in bovine milk. While 95% of the casein content is naturally self-assembled into casein micelles, i.e., spherical colloidal particles, approximately 10% is present in the form of single molecules as soluble casein. These two states, *viz.* molecular and micellar casein, exist in equilibrium [17]. Caseins can form dual-binding models that exploit their amphiphilic nature; interactions exist between hydrophobic regions of the proteins, with calcium phosphate clusters being linked to phosphoseryl clusters [18].

#### 2.1.1. Biological Properties of Casein

**Figure 1:**
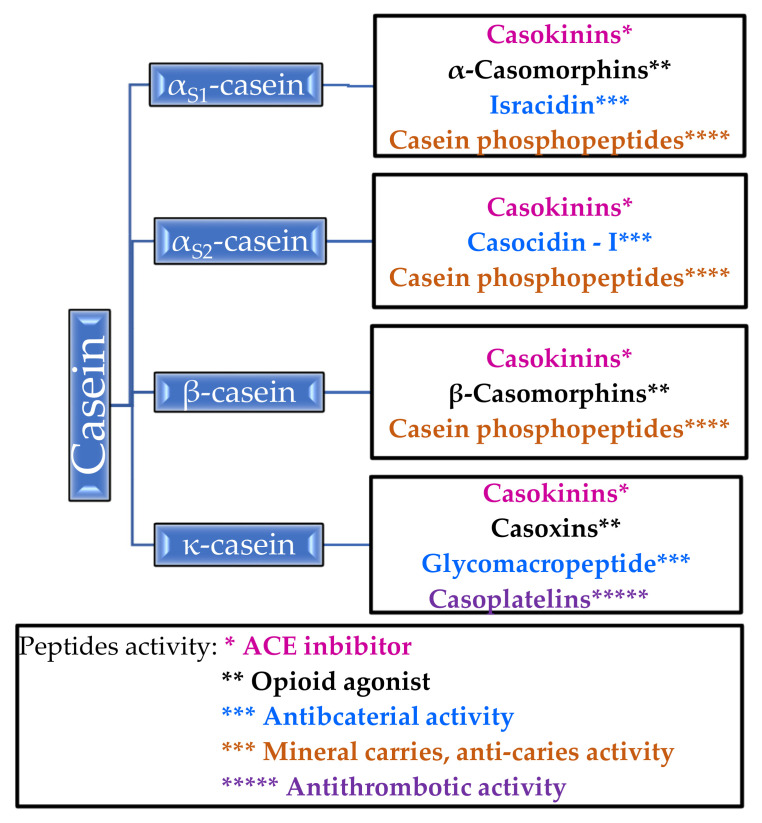
Peptides released from casein and their activity.

The phosphoproteins contained in casein regulate the calcium and phosphate metabolism in the body. Improvement in bone mineralization in experimental animals was observed in postmenopausal models [19]. In addition, casein proteins inhibit tooth decay by increasing the calcium phosphate content in the plaque [20] (Table 2). α-Casein, β-casein, αs1-casein, αs2-casein and κ-casein can transform into biologically active molecules. For example, α-casein forms an opioid, casomorphine, and α- and β-caseins are precursors of immunopeptides. In Figure 1, the bioactive peptides that are released from casein and their properties are shown [14,16,21].

**Table 2:** Major bioactive protein components of bovine, goat, sheep colostrum, and milk.

Proteins	Colostrum			Milk			[Ref]
	Bovine	Goat	Sheep	Bovine	Goat	Sheep	
Casein (g/L)	2.6	n.d.	n.d.	* 2.7; 2.8	2.5	4.6	[14], * [22]
κ-casein (%)	n.d.	n.d.	n.d.	* 12	** 20.4	* 9.1–10.2 ^a^	* [23,24], ** [22]
αS1-casein (%)	n.d.	n.d.	n.d.	* 37	** 5.6	* 33.9–39.9 ^a^	* [23,24], ** [22]
αS2-casein (%)	n.d.	n.d.	n.d.	* 10	** 19.2	* 12–16.4 ^a^	* [23,24], ** [22]
β-casein (%)	n.d.	n.d.	n.d.	* 35	** 54.8	* 37–42.3 ^a^	* [23,24], ** [22]
β-lactoglobulin (mg/mL)	* 7.9–30	* 9.3–49.8	** 4–19	*** 3.3	**** 3.07	**** 5.97	* [25], ** [26], *** [14], **** [27]
α-lactalbumin (mg/mL)	* 3	** 2.77	*** 1.5–2	* 1.2	**** 1.27	**** 0.95	* [14],** [25],*** [26],**** [27]
Immunoglobulins (g/L)	20–150	n.d.	n.d.	0.5–1	n.d.	n.d.	[14]
IgG	* 15–180	** 50–60	*** 45–69	* 0.35; ** 0.59 ^b^	** 0.1–0.4	**** 0.35–1.62	* [28,29,30,31,32,33,34], ** [22], *** [35], **** [36]
IgM	* 4.2; ** 5 ^b^	* 1.6–5.2	*** 1.3–21.20	* 0.05	* 0.01–0.04	*** 0.2	* [13,37],** [28,29,30,31,32,33,34],*** [38]
IgA	* 3.5; **3.9 ^b^	** 0.9–2.4	*** 3.5	** 0.14	** 0.03–0.08	*** 0.2	* [28,29,30,31,32,33,34],** [13,37],*** [38]
Glycomacro-peptide (g/L)	2.5	n.d.	n.d.	1.2	n.d.	n.d.	[14]
Lactoferrin (g/L)	* 0.8; ***1.5–5 ^b^	** 0.38	** 0.74	* 0.02–0.2; 0.02–0.75 ^b^	* 0.098–0.15	* 0.14	* [39,40,41,42], ** [36], *** [43]
Lactoperoxidase (g/L)	* 0.02; *** 0.011–0.045 ^b^	** 0.062–0.204	n.d.	* 0.03; *** 0.013–0.03 ^b^	n.d.	n.d.	* [14], ** [8], *** [6]
Lysozyme (mg/L)	* 0.14–0.7	n.d.	n.d.	** 0.37–0.6	** 0.25	** 1–4	* [44], ** [13]
Serum albumin (g/L)	* 1.3	n.d.	n.d.	* 0.3	** 0.26–0.3	** 0.55–0.6	* [14], ** [45]
Growth factors (µg/L)	50 µg–40 mg/L	n.d.	n.d.	<1 µg–2 mg/L	n.d.	n.d.	[14]
IGF-I	* 0.049–2 ^a^	n.d.	*** 0.199–0.265; ** 50–500 ^b^	* <0.002–0.101	**** 11–16.8 ^a^	** “low”	* [46,47,48,49,50,51,52,53,54,55,56,57,58],** [59],*** [60],**** [61,62,63]
IGF-II	** 0.15–0.6 ^a^	n.d.	n.d.	** 0.002–0.1 ^a^	* 106	n.d.	* [62], ** [46,51,56]
EGF	* 0.004–0.008; 0.3242 ^b^	n.d.	** 1.7–2.3	<0.002; 0.155 ^b^	n.d.	** <0.0008	* [64,65], ** [66]
TGF-β1	0.0124–0.0426	n.d.	n.d.	0.0008–0.0035	n.d.	n.d.	[49]
TGF-β2	0.15–1.15; 0.3 ^b^	n.d.	n.d.	0.013–0.07; 0.066 ^b^	n.d.	n.d.	[48,67]

n.d.: no data; ^a^ the minimum and maximum values from the test are given; ^b^ found different values; *, **, *** and **** refer to the corresponding [Ref] in the last column.

It is worth paying attention to casein phosphopeptides, which exhibit the properties of mineral carriers and anti-carriers—they form complexes with calcium ions and other mineral ions, thanks to which they increase the absorption of calcium in the intestines [14]. Unlike β-casomorphins, which are opioid agonists, casoxins are opioid antagonists [14,21]. Antithrombotic activity has been demonstrated for casoplatelins and antimicrobials in in vivo studies, especially against *Streptococcus aureus*, *Streptococcus pyogenes*, and *Listeria monocytogenes* [14]. Several of the listed peptides are described in more detail in Section 2.1.2.

#### 2.1.2. Properties of Casein Peptides

Casokinins are fragments of α-casein and β-casein; these are believed to exhibit antihypertensive effects by acting as angiotensin-I converting enzyme (ACE) inhibitors [9]. Casokinins are mainly produced by the action of trypsin or chymotrypsin on β- and α_S1_-casein [21]. ACE is an enzyme that catalyzes the conversion angiotensin I to the strong vasoconstrictor angiotensin II, thereby increasing blood pressure. Casokinins inhibit the action of ACE and thus lowers blood pressure in in vivo and in vitro studies [68,69,70].

Caseinomacropeptide (CMP) is split from casein by rennin during milk coagulation. It inhibits the aggregation of blood platelets and the binding of the human fibrinogen γ-chain to fibrinogen receptors on the platelet surface [14].

κ-casein is a donor of glycomacropeptide (GMP) and can be obtained by the action of chymosin [9]. GMP has been found to inactivate microbial toxins of *Escherichia coli* and *Vibrio cholerae*, inhibit the adhesion of cariogenic *Streptococcus mutans* and *Streptococcus sobrinus*, and hemagglutination by four strains of influenza virus in in vitro tests. It also modulates immune system responses, promotes the growth of *Bifidobacteria*, suppresses gastric hormone activities, and regulates blood circulation through antihypertensive and antithrombotic activity [71,72].

GMP hydrolysate (GHP) has been found to increase the level of hepatic glycogen and ameliorate hepatic insulin resistance in high-fat diet (HFD)-fed mice, suggesting that GHP may improve the insulin sensitivity of insulin target organs [73]. GHP could reduce the levels of interleukin-6 (IL-6), interleukin-1 beta (IL-1β), and tumor necrosis factor-alpha (TNF-α) in macrophages [74].

GHP has been found to demonstrate effective hypoglycemic activity and to ameliorate dyslipidemia and inflammation in diabetic mice. GHP supplementation could influence the composition and diversity of gut microbiota, with potentially beneficial effects on the insulin signaling pathway and host metabolism. Hence, GHP may support the prevention and management of type 2 diabetes [75].

#### 2.1.3. Casein as Nanocarrier for Some Drugs

As casein micelles act as carriers to provide newborns with nutrients such as calcium, phosphate, and protein, it has been proposed that they may be used to deliver vitamins, minerals, and antibacterial substances. Such drug delivery systems may improve the efficiency of drugs and avoid their toxic effects.

It has been shown that the micelles not only have an affinity for vitamin D2 but also protect it from light by absorbing its radiation on its surface, thus protecting drugs such as ergocalciferol from degradation [76].

In addition to its strong surface activity, particle stabilizing effect, and good adhesion strength, casein can form films; however, these films show poor flexibility and water resistance and are easily decomposed under the influence of bacteria, which limits their further use [77]. In order to improve the properties of casein, core-shell structural casein-based ZnO nanocomposites have been created by double in situ polymerization. The resulting nanocomposite film demonstrates improved thermal stability, lower water absorption rate, and excellent antibacterial properties against *E. coli* [77].

It has been found that β-casein also forms complexes with other biologically significant substances, protecting them and increasing their bioavailability. For example, binding resveratrol to β-casein in a casein micelle significantly improves its bioavailability. Both the existing cis- and trans-resveratrol isomers can be trapped in the micelle, and the beta-casein-resveratrol complex has a much better protective effect during storage than β-casein micelles [78].

A particularly interesting and innovative example of the use of casein as a carrier is the creation of a complex with platinum. The clinical application of platinum-based anticancer drugs is greatly limited by their severe toxicity; however, a platinum(II) complex of bipyridine morpholine dithiocarbamate with nanoparticles composed of β-casein and chitosan demonstrated improved cytotoxicity and cellular uptake against colorectal cancer HCT116 cells, suggesting that this novel drug delivery system could enable the drugs to function in stable aqueous solutions and to be of use in targeted oral delivery applications. The nanoparticles demonstrated good colloidal stability and low cytotoxicity [79].

### 2.2. α-Lactalbumin

α-Lactalbumin is a hydrophilic albumin and globular protein. A single peptide chain consists of 123 amino acids. It accounts for about 20% of bovine whey proteins [80]. This albumin is a coenzyme in lactose biosynthesis and has the function of transporting calcium metal ions [44].

#### Biological Properties of α-Lactalbumin

α-Lactalbumin is a good source of opioid peptides. It has the ability to reduce stress and depressive moods by increasing brain tryptophan and serotonin levels [81,82,83].

Native α-lactalbumin does not show antibacterial activity, unlike peptides isolated from α-lactalbumin: LDT1 (1–5), LDT2 (17–31) S-S (109–114), LDC (61–68), and S-S (75–80). The first two peptides are formed under the influence of trypsin, and the third by chymotrypsin [84]. They have been found to be active mostly against *Staphylococcus epidermidis* ATCC 12228, *Staphylococcus lentus*, and *Bacillus subtilis* BGA [84].

The HAMLET/BAMLET (human α-lactalbumin made lethal to tumor cells/bovine α-lactalbumin made lethal to tumor cells) complex with oleic acid can penetrate tumor and immature cells, interfering with mitochondria, nucleosomes, and proteosomes, and activating apoptotic cancer cell pathways [85,86]. It has been reported to have comparable cytotoxic activity against lung cancer, kidney, and bladder carcinoma cell lines [44,87,88]. These findings suggest that such cytotoxic aggregates of apo-alpha-lactalbumin could be potential antitumor drugs.

### 2.3. β-Lactoglobulin

β-Lactoglobulin belongs to whey proteins and constitutes about 50% of these proteins [89]. Its content in colostrum is much higher than in milk and amounts to 7.9–30 mg/mL [6]. Structurally, it is a globular protein containing five cysteine residues, four of which are involved in the formation of disulfide bridges stabilizing the quaternary structure [44]. β-Lg is a rich source of calcium ion binding peptides [90].

Regarding its thermal stability, a temperature of 72 °C did not cause significant changes in the structure of the β-Lactoglobulin molecule in mixtures of other substances derived from milk; however, heating for 30 s at 100 °C resulted in significant changes, i.e., partial denaturation of particles [91]. This globulin is a carrier of retinol and fatty acids and is known to bind vitamin D and stimulate lipase activity [40,41,42]. In addition, when heated to 70–80 °C, it loses the ability to actively bind palmitic acid, vitamin D, and retinol [92].

#### Biological Properties of β-Lactoglobulin

Hernandez-Ledesma et al. isolated several antioxidant peptides by hydrolysis with Corolase PP. Their antioxidant activity was slightly higher than that of butylated hydroxyanisole (BHA) [93].

The β-Lg molecule also demonstrates significant antioxidant activity in milk, which, among others, has been attributed to the presence of sulfur-containing amino acids such as methionine [89,90,91]. These amino acids are also believed to exert antitumor effects [94]. Their activity is believed to be associated with the fact that methionine is a precursor of cysteine, which is needed for the formation of glutathione (GSH): a thiol antioxidant that scavenges reactive oxygen species, resulting in the formation of oxidized glutathione. Decreased amounts of GSH and a decreased GSH/GSSG ratio in tissues are biomarkers of oxidative stress. Chronic oxidative stress may lead to chronic inflammation and cancer development and progression [95].

The protein demonstrates antimicrobial effects by inhibiting the adhesion of pathogens to surfaces and thus preventing their colonization [80,96]. Bactericidal activity has been shown against both Gram-positive bacteria, such as *B. subtilis* and *S. aureus*, and Gram-negative ones, such as *E. coli* and *Bordetella bronchiseptica* [80]. Other studies also indicate that β-Lg chemically modified with 3-hydroxyphthalic anhydride, may be effective in inhibiting *Chlamydia trachomatis* infection; in addition, 3-HP-β-lactoglobulin is active against herpes simplex virus HSV-1 and -2 [97].

### 2.4. Lactoferrin

One of the bioactive whey proteins is Lactoferrin (LF). It was first isolated in 1939 from cow milk and later from human milk in 1960 [98]. It demonstrates a similar iron-binding capacity to transferrin proteins [44]. Lactoferrin is a monomeric glycoprotein; its polypeptide chain consists of two spherical lobes connected by a hinge region [99].

It is resistant to high temperatures and proteolytic enzymes [100]. LF can be found in saliva, bile, pancreatic fluid, amniotic fluid, and tears, but the highest concentration is found in human or porcine milk [44]. It is a component of neutrophils, from which it is released into the bloodstream during trauma, infection, and inflammation [101,102,103].

In milk, LF is mainly synthesized by glandular epithelial cells [103]. Its concentration ranges from 20 to 200 mg/L in cow milk, 140 mg/L in sheep milk, and 98–150 mg/L in goat milk [13,39,40]. Higher levels are found in the colostrum than in milk; for example, the level is around 0.8 g/L in cow colostrum [41].

#### 2.4.1. Biological Properties of Lactoferrin

Lactoferrin is a protein that is found in many body fluids such as colostrum, milk, tears, nasal secretions, saliva, and genital secretions. It is also produced in large quantities in neutrophils [42]. Lactoferrin demonstrates bactericidal, bacteriostatic, antiviral, antifungal, antiparasitic, anticancer and antioxidant properties [41,100,104,105,106,107,108,109,110,111,112,113,114]. In addition, several clinical studies have confirmed that bovine lactoferrin is an immune modulator that stimulates the phagocytic activity of multinucleated leukocytes [107] and reduces the production of interleukin (IL)-6 and tumor necrosis factor (TNF)-α in cell cultures [106]. The mechanisms of action of lactoferrin related to individual types of its activity were presented in Table 3.

The protein exerts antibacterial activity by chelating iron and removing it from the microbial growth environment [128]. It is also involved in the direct destruction of the sheaths and disruption of bacterial cell metabolism by inhibiting adhesion to host tissues [129], inhibition of biofilm formation by some bacteria [130], and stimulating the immune system to fight pathogens [112].

It has been proven to have a protective effect on intestinal epithelial cells and on the growth of beneficial intestinal microflora while inhibiting the growth of pathogenic bacteria such as *E. coli*, and especially those of the Enterobacteriaceae family [108].

It should be mentioned that the glycoprotein increases the sensitivity of bacteria to certain antibiotics such as vancomycin or penicillin, which reduces the supply of effective doses of individual drugs [131]. Diarra et al. showed that a mixture of lactoferrin and penicillin doubled the inhibitory activity against *S. aureus* [131].

Van der Kraan et al. isolated and characterized a new peptide, lactoferrampin, which, together with lactoferricin, a peptide derived from the hydrolysis of lactoferrin, showed antimicrobial properties against *E. coli*, *L. amonocytogenes*, *B. subtilis*, *Pseudomonas aureoginosa*, and *Candida albicans* [110].

Many clinical studies have found lactoferrin to have antiviral properties. It effectively inhibits the development of infection caused by hepatitis B and C [132], type I and II herpes simplex virus [128,133], HIV [134], human cytomegalovirus, HPV virus, enterovirus, influenza virus, and parainfluenza virus, and rotavirus [109,111,127,135].

The protein also prevents the formation of free radicals regulating the production and release of cytokines and tumor necrosis factor (TNF) [101]. Lactoferrin can serve as an antioxidant by sequestering cationic iron and copper and thereby inhibiting the propagation of hydroxyl radicals [136]. Lactoferrin has potential antioxidant properties due to the ability to sequester free iron ions [137].

### 2.5. Lactoperoxidase (LPO)

LPO is a glycoprotein that occurs naturally in milk, colostrum, and many other secretions [14]. It catalyzes the oxidation reaction of thiocyanates in the presence of hydrogen peroxide, thereby generating intermediates with a broad spectrum of antimicrobial activity [138,139,140]. Lactoperoxidase acts as a natural antibacterial agent as an element of non-specific cellular immunity [100,141]. Its concentration is 13–30 mg/L in cow colostrum and 11–45 mg/L in milk [6]. In vitro studies showed that LPO has bactericidal activity against Gram-negative bacteria such as *E. coli*, *Salmonella* spp., *Pseudomonas* spp., *Campulobacter* spp., and bacteriostatic properties against Gram-positive ones such as *Listeria* spp., *Staphylococcus* spp., *Streptococcus* spp. It has also demonstrated activity against *Candida* spp. and the protozoan *Plasmodium falcipari*, and has been found to inactivate HIV type 1 and poliovirus [142,143,144,145,146].

### 2.6. Immunoglobulins

They are high molecular globulins that can be divided into five classes, i.e., IgG, IgA, IgM, IgE, and IgD. Each class has a similar structure composed of four polypeptide subunits [147]. In bovine milk and colostrum, the primary immunoglobulins are IgG, whereas in human milk, 90% of all immunoglobulins are sIgA; therefore, there are differences in the specifics of the action of total immunoglobulins [148]. The protective effects of immunoglobulins are presented below, as exemplified by Igs from bovine milk and colostrum. It is believed that the Igs in milk transport immunity from the mother to the neonate [44]. By binding antigens and stimulating phagocytosis or activating the complement system, immunoglobulins prevent pathogen adhesion, inhibit bacterial metabolism, and also neutralize toxins. Immunoglobulins thus participate in the destruction of pathogenic microorganisms such as *E. coli*, *C. albicans*, *Clostridium difficile*, *Shigella flexneri*, *S. mutans*, *Helicobacter pylori*, and *Cryptosporiadium pravum* [149,150].

The physical properties of Igs have a key influence on their natural antimicrobial properties and the possibilities of their further use. Igs are thermolabile, especially at high temperatures. Heating milk at 100 °C for 30 s damages the structure of Ig proteins, while heating at 72 °C for 15 s allows for mild pasteurization and maintains their active properties with no structural changes [91]. Therefore, extensive use of immunoglobulins is possible.

### 2.7. Lysozyme

Lysozyme is a hydrolase found at high concentrations in tears and chicken egg whites, from which it is obtained on an industrial scale, mainly by direct crystallization. Other methods for its preparation, like sequential dilution diafiltration using a UF membrane, affinity chromatography, adsorption, or molecular imprinted particles (Lys-MIP), are also known but used only in laboratory practice due to high costs [151,152]. Lysozyme stimulates the non-specific humoral immune response [141,153]. Its content has been found to vary from 0.37–0.6 mg/L in cow milk, 0.25 mg/L in goat milk, to 1–4 mg/L in sheep milk [13], and to range from 0.07–0.6 mg/L cow milk and 0.14–0.7 mg/L in colostrum [44].

Lysozyme exerts its antimicrobial activity by catalyzing the hydrolysis of the β-1,4 bonds in peptidoglycan, a component of bacterial cell walls; it is active against Gram-positive bacteria and demonstrates synergistic bactericidal activity with lactoferrin against *S. epidermis* [44]. Lysozyme is also called an endogenous antibiotic due to its supportive effect on bactericidal and bacteriostatic drugs [105]. The formation of a complex of lysozyme with oleic acid shows promising bactericidal effects against *Streptococcus pneumoniae* [154], and the lysozyme-ZnO nanoparticles complex demonstrates synergistic activity against *E. coli* and *S. aureus* [155]. Lysozyme applications in the industry could expand with the increased production of lysozyme afforded by the use of transgenic animals, whose milk contains levels as high as 25 mg/L [44].

### 2.8. PRP (Proline-Rich Peptide)

It is a complex composed of a mixture of 32 peptides of various molecular masses ranging from 500–3000 kDa. This group of similar molecules demonstrates a broad spectrum of regulatory activity supporting the development of the immune system, inducing the maturation and differentiation of murine thymocytes, and affecting humoral and cellular immune responses. PRP consists of proline residues (25%) and hydrophobic amino acids (40%) [156]. It possesses homology to three protein precursors: annexin, β-casein, and a hypothetical β-casein homolog [157].

PRP was first isolated from ovine colostrum and was subsequently found in human and cow colostrum [156]. PRP exhibits immunomodulatory properties, inducing maturation and differentiation of thymocytes, the proliferation of pheochromocytoma cells increases the viability of fibroblast cells and inhibits β-amyloid-induced apoptosis [158]. It has been found to influence precognitive functions in animal models, and hence to exert a potential influence on central nervous system processes. Clinical studies on the effects of sheep colostrum PRP administration in patients with Alzheimer’s disease (trade name Colostrinin Tm; ReGen Therapeutics Ltd., London, UK) found it to have a beneficial effect on disease symptoms and everyday functioning in AD patients and a negligible number of very mild side effects [159].

### 2.9. Growth Factors

Growth factors, which are generally considered a subset of cytokines, are signaling proteins that stimulate cell growth, differentiation, survival, inflammation, and tissue repair. They can be secreted by neighboring cells, tissues and glands, and even cancer cells. All cells need a range of growth factors to maintain proliferation and viability [160,161,162]. The first information about the presence of growth factors in milk and colostrum was recorded in 1997 by Pakkanen and Aalto and later by Gauther et al. in 2006 [141,163]. The following growth factors are known to be present in milk: BTC (Betacellulin GF), EGF (Epidermal GF), FGF-1, FGF-2 (Fibroblast GF), IGF-1 and IGF-2 (Insulin-like GF), TGF-β1, TGF-β2 (Transforming GF), and PDGF (Platelet-derived GF) [101]. Of these, the most prevalent are epidermal (EGF), insulin-dependent (IGF), and transformative (TGF) growth factors [141], with concentrations of 2–155 ng/mL for EGF, 2–101 ng/mL for IGF-I, 2–107 ng/mL for IGF-II and 13–71 ng/mL for TGF-β2, and <4 ng/mL for BTC, TGF-β1, FGF1, and FGF2 [163].

Growth factors demonstrate a multilevel effect on individual cells and tissues. EGF and BTC stimulate the proliferation of epidermis, epithelium, and embryonic cells, and promote wound healing, bone reconstruction and inhibit gastric secretion [163]. TGF-β is responsible for embryo development and repair and the formation of bone, cartilage, and epithelial tissue; it also regulates the immune system [163]. Some supplements have been designed with the aim of increasing immunity and treating conditions such as childhood Crohn’s disease, e.g., Nestle Modulen IBD containing TGF-β2 from milk [164,165,166]. IGF, which includes IGF-I and IGF-II, stimulates the proliferation of many cells and also regulates metabolic functions, e.g., glucose uptake or glycogen synthesis [163]. Due to their wide range of action, these growth factors have been included in studies on treating epilepsy [167]. Playford et al. [168] suggest that cow colostrum-based products that contain growth factors can be used to counteract side effects when using nonsteroidal anti-inflammatory drugs. Gauthier et al. [163] described a positive effect of growth factors on the digestive system, skeletal system, and skin.

Particularly important in the context of this article seems to be the study, the results of which indicate that FGF, IGF-1, and EGF are important mitogens in wound healing and that EGF, in particular, is capable of stimulating the epithelium. IGF-1 and EGF may play a significant role in both the early and late wound environment, while FGF may play the most important role in early tissue repair [169].

## 3. Milk- and Colostrum-Based Products in Cosmetics and Dermatology

Recent years have seen a growth in interest in natural products, and milk- and colostrum-based products are now widely used in the cosmetics and pharmaceutical industries. In this chapter, their impact on skin conditions when used as a dietary supplement or topically applied in the form of creams, ointments, etc., will be discussed. These data are summarized in Table 4. This section will also present the effect of milk or milk-derived ingredients on skin cells (fibroblasts and/or keratinocytes) in vitro.

### 3.1. Impact of Supplementation with Milk- and Colostrum-Based Products on Skin Conditions

Substances derived from colostrum and milk, especially proteins, have been shown to have great therapeutic potential in the treatment and prevention of many diseases, and there is a growing demand for those based on colostrum. A number of colostrum-supplemented powders, capsules, lozenges, beverages, and chewing gums are available on the market. The products are used not only as wound healing factors and antioxidants, anti-inflammation, tissue growth agents but also to enhance the immune system, repair damaged gastrointestinal tissues, or encourage the differentiation and proliferation of epidermal cells, among others [5,43].

The milk protein most commonly used as a supplement is lactoferrin, possibly due to its broad spectrum of proven biological properties (Section 2.4.1). This has been found to induce a significant improvement in the skin condition of patients with psoriasis and acne vulgaris, including a reduction in the number of inflammatory lesions and an overall improvement in the clinical picture [90,170,171,172].

One study compared the effect of consuming fermented milk enriched with lactoferrin (200 mg daily) by patients with acne vulgaris in the course of skin inflammation. Two groups of 18 patients, one consuming enriched milk and the other unenriched milk (placebo), were tested for skin hydration, sebum, pH, and skin surface lipid content at the beginning of the study and after 12 weeks. The group of patients taking lactoferrin-enriched milk demonstrated a 38.6% improvement in inflammation reduction, 31.1% lower sebum content, 23.1% fewer total lesions, and 20.3% lower acne severity compared to the placebo group. Although both groups displayed a decrease in lipid level on the skin surface, the lactoferrin-receiving group also demonstrated a decrease in the triacylglycerol content of the lipids; this was found to correlate with the reduction in acne lesions and the severity of acne. The hydration and pH of the skin reminded unchanged after supplementation [90].

Twice daily administration of lactoferrin (100 mg) as a dietary supplement was found to result in an overall improvement in acne lesions in patients with mild to moderate common acne [170]. Twice daily administration of capsules containing lactoferrin with vitamin E and zinc for three months was found to reduce the number of acne lesions, reduce blackheads and inflammatory changes, and better regulate sebum secretion. The preparation was found to be both safe and effective [173].

In addition, an orally administered preparation based on milk proteins, rich in growth factors, alpha-lactalbumin, lactoferrin, and immunoglobulins in reducing skin lesions has been found safe for use in patients with plaque psoriasis [171].

Lactoferrin supplementation was also found to inhibit the increase in transepidermal water loss, reduction in skin hydration, aberrant epidermal hyperplasia, and cell apoptosis in hairless mice orally administered lactoferrin and exposed to UVB radiation [172].

The vitamins, minerals, and amino acids contained in colostrum are known to bestow many health-promoting effects on human skin. Ascorbic acid (vitamin C) is involved in the production of collagen and L-carnitine; it also maintains healthy skin, heals wounds, and possesses antioxidant activity. In addition, niacin (vitamin B3) maintains healthy skin, biotin (B7) strengthens hair and nails, vitamin E possesses antiaging and antioxidant activity, and retinol (vitamin A) encourages skin cell production and has antiaging properties. The various minerals present, such as zinc and copper, take part in neutralizing the harmful effects of free radicals, regeneration processes, and wound healing.

**Table 4:** Clinical studies with milk-or colostrum-based products.

Product Used	Type of Disease or Healthy Skin (Number of Patients)	Result of the Study	[Ref.]
Topically applied milk-based products			
bovine colostrum preparation (supported antibiotic therapy)	difficult-to-heal wounds caused by buttock erythematosus and by erosion erythema	significant improvement in wound healing	[174]
ointments containing 10% and 20% lactoferrin	moderate psoriatic plaque (*n* = 22)	improvement in elevation, redness, and scaling of psoriatic lesions	[114]
soap containing 5% Podolian cow milk	healthy skin	good cleansing and antibacterial properties	[175]
creams with skimmed donkey milk encapsulated in nanoliposomes	healthy skin (*n* = 15)	satisfactory moisturizing properties; antiaging effects	[176]
cream containing 30% horse colostrum	seborroic acne (*n* = 12)	complete skin regeneration	[177]
cream containing 20% horse colostrum and 10% horse milk (plus mint and benzocaine)	contact skin lesions (*n* = 5)	resolution of contact skin lesions and pain immediately after application	[177]
cream containing 20% horse colostrum and 10% horse milk (plus mint and benzocaine)	hyperthermia sunburn skin (*n* = 30)	immediate relief of pain and skin tension (within 24 h); the appearance of a normal tan, without any scale-off skin effect	[177]
cream containing 20% horse colostrum and 10% horse milk (plus mint and benzocaine)	2° degree and 3° fire burns (*n* = 8)	rapid pain relief; rebuilding the epithelium in a week	[177]
emulsion with 20% horse colostrum	moderate atopic dermatitis (*n* = 7)	reduction in erythema and pruritus; softening, moisturizing, soothing, and anti-inflammatory effects	[177]
liposomal gel containing 20% horse colostrum	ulcerative skin lesions (*n* = 10)	improvement of skin healing and repair	[177]
cosmetic formulations based on a combination of horse colostrum and horse milk	healthy skin	antiaging, moisturizing, protective, tensio-distensive, tonic, smoothing, anti-irritant, emollient, bleaching, decongestant, and sebostatic effects	[177]
fermented (by lactic acid bacteria) horse colostrum	atopic dermatitis (atopy and psoriasis)	alleviating symptoms; moisturizing and anti-inflammatory effects	[178]
fermented colostrum	acne	improvement related to the antibacterial effect	[179]
formulations containing bovine or equine colostrum (plus hyaluronic acid or its salt and olive oil or vitamin E)	healthy skin of elderly volunteers	improvement elasticity and tension; moisturizing and antioxidant effects; reduction in skin sagging and liver spots	[180]
cosmetic formulation based on colostrum albumin (plus arbutin)	healthy skin with discoloration	whitening properties	[181]
Milk-based products used as supplements			
fermented milk enriched with lactoferrin	acne vulgaris (*n* = 18)	reduction in inflammation, sebum content, and the severity of acne lesions	[90]
lactoferrin	mild to moderate common acne	overall improvement in acne lesions	[170]
capsules containing lactoferrin (plus vitamin E and zinc)	acne	reduction in the number of acne lesions, blackheads, and inflammatory changes; regulation of sebum secretion	[173]

The amino acids also have positive effects: proline has antiaging properties (reduces wrinkles and sagging), forms collagen, and heals tissue, threonine produces collagen and elastin in the skin, methionine has an antioxidant effect, synthesizes collagen in nails and hair and arginine stimulates wound healing [43].

### 3.2. Influence of Milk or Milk-Derived Ingredients on Skin Cells In Vitro

Skim bovine colostrum has been found to increase canine skin fibroblast proliferation. Bovine colostrum stimulated fibroblast growth at all doses (0.1, 0.3, and 1 mg/mL) after 24 h incubation. Proliferation was found to increase from 19% to 32% compared to negative controls, and the effect remained significant after 48 h for the 0.3 and 1 mg/mL doses [182].

The fat fractions isolated from mare’s colostrum were found to have a stronger effect on fibroblast proliferation in vitro than those from milk. The different lipid pattern of the two substances, specifically the higher levels of adipophilin and lactadherin in colostrum fat globules, is believed to have affected skin wound repair efficiency. Colostrum also contains higher levels of total lipids, linoleic and linolenic acids, gangliosides, and glycolipids when compared to milk [5,183].

Colostrum has a positive effect on the healing process of skin wounds. This may be due to the participation of growth factors and/or other immune regulatory factors [5].

Peptides from milk protein hydrolysates, typically with a molecular weight of 800 Da and containing mainly hydrophobic aromatic amino acids, have been found to promote growth in human skin cells in vitro. Treatment promoted growth efficacy by 108% in keratinocytes cultured in a medium supplemented with 300 μg/mL of one peptide fraction for 12 days [184].

Other studies have examined the effect of donkey colostrum and mature milk, human colostrum and mature milk, and β-casein and β-casomorphine-7 on the growth and inflammatory response of the skin fibroblast culture. Exposure of skin fibroblasts to donkey milk and human colostrum resulted in a decrease in proinflammatory transcriptional factor NF-κB p65 activity. The opposite effect was noticed for β-casein and β-casomorphine-7. Moreover, it was proved that the tested products and β-casein lead to the activation of growth-regulating kinases (Akt 1/2/3 kinase, Erk kinase, INK kinase, and Stat-1 kinase), especially the p-Erk pathway. Accordingly, it can be concluded that casein amino acids may be responsible for the activation and proliferation of the cell cycle initiated by Erk. It suggests that noncasein bioactive peptides of donkey and human milk may be responsible for anti-inflammatory properties and may be useful in wound healing, regenerative, and aesthetic dermatology [185].

Recently, Kovacs et al. showed that colostrum promotes cell cycle withdrawal by increasing the expression of kinase inhibitors and promotes the transition of keratinocytes from proliferation to differentiation. Colostrum also has the ability to induce the expression of early and late differentiating markers (keratin 1, involucrin, and filaggrin) and the synthesis of caspase 14 and bleomycin hydrolase: two major enzymes involved in the maturation of filaggrin. Bovine colostrum has been found to promote keratinocyte section and final differentiation in two-dimensional (2D) and three-dimensional (3D) skin counterparts, the latter being a more physiologically representative system. Colostrum appears to stimulate cell differentiation via the PI3K/PLC-γ1/PKCα (3-phosphatidylinositol kinase/phospholipase Cγ2/protein kinase Cα) pathways associated mainly with tyrosine kinase receptors; this suggests that colostrum may be used in the treatment of skin diseases characterized by a perturbed barrier function, such as cutaneous dryness in elderly or UVR-exposed subjects [186].

### 3.3. Topical Applications of Milk or Colostrum Containing Products

The properties of milk proteins make them promising candidates researchers are trying to use them to create a skin substitute for the treatment of burn wounds. A preliminary study showed that the application of bioactive milk proteins, lactoferrin, and whey proteins on a synthetic polymer (polycaprolactone) scaffold increased the growth, spread, and infiltration of keratinocytes and fibroblasts. Hence, it could be effectively used to heal wounds [187].

Milk proteins have also been used directly on the skin for therapeutic purposes. Topical application of bovine colostrum preparations resulted in significant improvement in the treatment of difficult-to-heal wounds caused by buttock erythematosus and by erosion erythema when administered as support for systemic antibiotic therapy [174].

An ointment consisting of *Gundelia* (*Gundelia tournefortii* L.) extract with milk cream (GT/MC 4:1) was found to support the healing of second-degree burn in a rat model. Thirty-six male Wistar rats with second-degree burns on the skin were divided into three groups: one treated with silver sulfadiazine drug, another treated with *G. tournefortii* L. extract composite with milk cream, and one untreated group. Of the three, the GT/MC ointment group demonstrated the most effective healing. After 21 days of treatment, the wound area was reduced, and the wound healing process was improved significantly. Thus, such an ointment could aid in the healing of burn wounds [188].

Bovine lactoferrin may be considered as a topical treatment option for the treatment of psoriatic plaque. This is confirmed by studies conducted on a group of 22 patients with moderate plaque psoriasis who used ointments containing 10% and 20% lactoferrin. The psoriatic lesions treated with lactoferrin demonstrated improvements in elevation, redness, and scaling; however, the 20% ointment did not appear to be more effective than the 10% [114].

Podolian cow milk has also been used as a raw material for skincare products in the form of liquid hand soap. The product was found to demonstrate good cleansing and antibacterial properties. The most effective was the soap containing 5% of Podolian milk, which reduced bacterial hand contamination by 98%. The antibacterial activity of the product derived from the presence of some milk protein, as well as various enzymes, such as lysozyme (0.25 mg/L), lactoferrin, and lactoperoxidase [175].

Another study compared the effects of creams with nanoliposomes encapsulated with skimmed donkey milk with placebo on untreated skin in fifteen healthy volunteers. Shortly after application, the creams demonstrated satisfactory moisturizing properties, which were maintained throughout the entire period. Transepidermal water loss slightly decreased only after four weeks, and the pH value was similar after each of the treatments. The creams may contribute to additional antiaging effects [176]. The formulations containing the skimmed donkey milk are described in detail in the Serbian patent (P-2016/0289; No 57752) [189].

Scientific publications regarding the external application of colostrum to the skin are sparse. However, many patents relating to colostrum-based dermocosmetics are pending.

In accordance with the patent US5750149A [177], formulations containing horse colostrum together with horse milk have been clinically tested. The studies investigated preparations with different ratios of colostrum to milk content, i.e., 30% horse colostrum, 20% horse colostrum and 10% horse milk, 10% horse colostrum and 20% horse milk, and 3% horse colostrum and 20% horse milk. When applied to the skin, the formulations were effective in the treatment of sunburns, burns from fire, contact lesions, skin diseases such as acne, seborrhea dermatitis, irritation, keratosis defects (psoriasis and ichthyosis), including atopic dermatitis and itching, among others [177].

In addition, twice-daily topical application of a cream containing 30% of horse colostrum led to resolution of cutaneous lesions in twelve patients aged 16–27, affected by seborrheic acne, with the skin being completely regenerated [177].

Twice-daily topical treatment, i.e., every 12 h, with a cream containing 20% horse colostrum and 10% horse milk, with the addition of mint and benzocaine, resolved contact skin lesions within 24 h in five test subjects, with the pain disappearing immediately after application [177]. The same cream, based on 20% horse colostrum and 10% horse milk plus mint and benzocaine, was applied twice-daily in adult patients with hyperthermia sunburn skin; the same extract was also applied to children but with the addition of chamomile extract instead of peppermint. The study group consisted of thirty people. The results indicated that pain subsided immediately, skin tension dropped over the course of 24 h, and a normal tan appeared, without any scale-off skin effect in the following days [178]. The cream was also found to be effective in the treatment of 2° degree and 3° fire burns. Similarly, in a group of eight patients, pain weakened quickly, and the epithelium was restored within one week [177].

Application of 20% horse colostrum emulsion (twice a day for 30 days) resulted in greater softening, moisturizing, soothing, and anti-inflammatory effects than observed for commonly available emulsions in seven patients with moderate atopic dermatitis compared to a control group of 10 healthy people. No side effects were observed, and erythema and pruritus decreased [177].

A liposomal gel containing 20% horse colostrum resulted in total or partial improvement of skin healing and skin repair in ten patients with ulcerative skin lesions treated twice daily for 20 days [177].

Irrespective of the composition, cosmetic formulations based on a combination of horse colostrum and horse milk demonstrate a number of skin benefits, including antiaging, moisturizing, protective, tensio-distensive, tonic, smoothing, anti-irritant, emollient, bleaching, decongestant, and sebostatic activities [177].

Another interesting patent study concerns the use of fermented horse colostrum in the case of atopic dermatitis. In this case, the colostrum was treated with lactic acid bacteria: *Lactobacillus delbrueckii subsp. bulgaricus* (ATCC 11842), *Streptococcus thermophilus* (ATCC 19258), and *Lactobacillus rhamnosus GG*, (ATCC 53103). The results indicate excellent moisturizing and anti-inflammatory properties, suggesting that the substance in question can be used to alleviate symptoms associated with atopic dermatitis, such as atopy and psoriasis [178].

A Korean patent describes the use of fermented colostrum as an anti-acne treatment. The specific active substance contained in colostrum, resulting from fermentation, appears to demonstrate excellent antibacterial effects against acne bacteria [179].

Another product containing bovine or equine colostrum, hyaluronic acid or its salt, and other substances such as olive oil (*Olea europea*) or vitamin E was found to reduce skin sagging, improve skin elasticity and tension, and demonstrate moisturizing and antioxidant properties when applied to the facial skin of elderly participants. Treatment also appeared to reduce the appearance of liver spots to a slight degree [180].

One patent presents a method of producing whitening cosmetics in which colostrum albumin is combined with arbutin, a whitening agent [181].

## 4. Conclusions

The broad spectrum of biological properties of milk and colostrum is undoubtedly related to their rich composition, especially in whey proteins, such as α-lactalbumin, β-lactoglobulin, lactoferrin, lactoperoxidase, lysozyme, immunoglobulins. Milk proteins enjoy unflagging interest among medical scientists not only because of their activities but also because of their natural origin and non-toxicity. These ingredients are considered in treating skin diseases, restoring immunity, fighting cancer, and combating microorganisms. Despite the multitude of studies on milk raw materials, there are few clinical trials confirming their effectiveness. Milk-based products appear to be used more often as dietary supplements than as medications.

It is also important to note that differences in the composition of milk between animal species may influence their therapeutic value. As bovine milk has been quite exhaustively studied, future research should be directed towards the milk from other species: sheep, goats, or camels.

Among milk raw materials, colostrum deserves special attention. Its complex composition has been found to be safe for use in people and to give excellent results in rebuilding immunity, as well as its use as a therapeutic agent in dermatology and an active ingredient of cosmetics. Topically applied formulations containing colostrum support healing processes of burn wounds, ulcers, and sunburns. They also appear to be effective at treating skin diseases, such as acne vulgaris, plaque psoriasis, and contact lesions. However, most research concerns the synergistic effects yielded by the combination of milk-derived ingredients/products and other active substances, like vitamin E, zinc, arbutin, hyaluronic acid, which does not allow for unequivocal conclusions about the action of the milk-based products themselves. Therefore, clinical trials are needed on a separated from a single milk ingredient or milk/colostrum products without any other active additives.

## Figures and Tables

**Table 3:** Mechanisms of lactoferrin action.

Kind of Activity	Mechanism of Action	[Ref.]
Antibacterial	- Reducing the concentration of iron ions that are necessary to bacterial growth and proliferation (chelation of iron via LF) - Interacting with lipoteichoic acid (LTA) of the cell walls of G(+) bacteria, disintegrating them and increasing their permeability	[102,115,116]
	- Binding to lipopolysaccharide (LPS) of the walls of G(−) bacteria and disintegrating them.	
Antifungal	- Damaging cell membranes of fungi and altering their permeability	[117,118,119]
	- Sequestration of iron	
	- Membrane destabilization	
Antiviral	- Blocking the host’s cell surface receptors due to the LF’s affinity for glycosaminoglycans- Direct interacting with capsid or viral envelope proteins	[113,120,121]
Antiparasitic	- Targets the host cell entry	[122,123,124,125,126]
	- Sequestration of iron- Probably linked to sequestration of iron	
	- Acts additively or synergistically with the antiparasitic compounds used in therapy	
Antioxidant	- Inhibiting the propagation of hydroxyl radicals by sequestering cationic iron and copper	[109,115,127]
Anticancer	- Reducing the production of tumor necrosis factor (TNF)-α in cell cultures	[104]
Immunomodulatory	- Stimulating the phagocytic activity of multinucleated leukocytes	[98,103,104]
	- Reducing the production of interleukin (IL) -6 in cell cultures	
	- T-cell maturation	
	- Stimulation of NK (natural killer cells) cells	
	- Reducing pro-inflammatory cytokines	
